# Reduction of surgical-site infections and improvement of scar parameters with a uniform fractional ultra-pulse CO₂ laser protocol in cosmetic surgery: a five-center retrospective cohort

**DOI:** 10.3389/fmed.2025.1642041

**Published:** 2025-11-21

**Authors:** Qinghua Xu, Haoran Li, Juhui Zhao, Xiao He, Liliang Zhao, Xiaofeng Zhang

**Affiliations:** Department of Medical Cosmetic Plastic Surgery, Central Hospital of Hanzhong, Hanzhong, China

**Keywords:** ultra-pulse CO₂ laser, cosmetic surgery, post-surgical infections, scar quality, laser

## Abstract

**Background:**

Postoperative infections and suboptimal scar outcomes remain persistent challenges in cosmetic surgery, impairing patient satisfaction and quality of life. This study evaluates whether a standardized fractional ultra-pulse CO₂ laser regimen can mitigate these complications.

**Methods:**

A multicenter retrospective cohort enrolled 562 adults undergoing elective rhinoplasty, liposuction, breast augmentation, facelift, abdominoplasty, or Botox. Laser eligibility (incision >3 cm, Fitzpatrick I–IV, no collagen-vascular disorder) was determined by an EMR-embedded checklist; 324 patients received a uniform three-stage laser protocol (pre-incision, immediate post-closure, day 10). All patients followed a five-day antibiotic regimen (oral cefalexin + topical mupirocin). Baseline covariates were balanced via IPTW. SSIs were microbiologically confirmed (aerobic, anaerobic, fungal, mycobacterial cultures with MALDI-TOF). Comparative analyses used Welch’s *t*-test and Fisher’s exact test; infection-free survival was assessed by Kaplan–Meier curves, and IPTW-adjusted Cox models quantified laser effects on SSI risk.

**Results:**

Of 562 patients, 324 (58%) received laser therapy. Baseline demographics and comorbidities were balanced across groups (all *p* > 0.05). Laser-treated patients had faster healing (13.8 ± 5.7 vs. 17.0 ± 3.2 days; *p* < 0.001), lower CRP (11.3 ± 7.4 vs. 14.9 ± 4.4 mg/L; *p* < 0.001), thinner scars (2.44 ± 1.39 vs. 3.14 ± 0.78 mm; *p* < 0.001), and greater elasticity (0.85 ± 0.03 vs. 0.65 ± 0.03 AU; *p* < 0.001). Pigmentation, vascularity, and collagen III/I ratios were all significantly improved (*p* < 0.001). SSIs were reduced (15% vs. 59%; *p* < 0.001), as were keloids (1.9% vs. 11%; *p* < 0.001). Satisfaction, quality-of-life scores, and return to activity favored the laser group (all *p* < 0.001). Kaplan–Meier curves confirmed superior 30-day infection-free survival (log-rank *p* < 0.001); IPTW-adjusted Cox regression showed a 72% reduced infection hazard (HR = 0.28; 95% CI: 0.19–0.41; *p* < 0.001).

**Conclusion:**

Standardized fractional ultra-pulse CO₂ laser therapy reduces SSIs, improves scar outcomes, and enhances patient satisfaction in cosmetic surgery. These findings support wider adoption of laser protocols, warranting further prospective, pathogen-specific research.

## 1 Introduction

Cosmetic surgery has experienced a notable increase in popularity over recent decades, driven by societal emphasis on aesthetic appearance and significant advancements in surgical techniques (1, 2). As the demand for procedures such as rhinoplasty, facelifts, and liposuction continue to rise, ensuring optimal postoperative outcomes becomes increasingly critical (1, 3, 4). One of the primary concerns in this context is the prevention of surgical site infections (SSIs), which not only hinder patient recovery and satisfaction but also impose substantial economic burdens on healthcare systems globally (5, 6).

Traditional infection control measures, including antibiotic prophylaxis and maintaining sterile surgical environments, have been fundamental in reducing SSIs (7–9). However, the growing prevalence of antibiotic-resistant pathogens and the limitations inherent in conventional methods highlight the need for innovative strategies to enhance infection prevention in cosmetic surgeries (9, 10). Among these emerging strategies, fractional ultra-pulse CO₂ laser treatment has garnered attention for its potential role in mitigating postoperative infections (11).

Fractional ultra-pulse CO₂ laser technology, initially developed for precise tissue ablation and hemostasis, has evolved to exhibit significant antimicrobial properties (12). Unlike conventional pulsed CO₂ lasers, fractional systems deliver energy in a grid-like pattern, creating microthermal zones that promote tissue regeneration while minimizing thermal damage. The antimicrobial efficacy of fractional CO₂ lasers is attributed to their ability to disrupt microbial cell walls, denature proteins, and interfere with DNA replication, thereby inhibiting pathogen proliferation (13–15). Additionally, specific laser wavelengths fractional CO₂ lasers have been shown to promote wound healing and modulate inflammatory responses, further contributing to improved surgical outcomes (16, 17).

Empirical studies across various surgical disciplines have demonstrated the effectiveness of fractional laser treatments in reducing SSIs (18, 19). For instance, studies reported a substantial decrease in infection rates in dermatologic surgeries when fractional diode lasers were employed for preoperative skin decontamination (20, 21). Similarly, research in orthopedic and dental surgeries has shown that fractional Nd and CO₂ lasers, respectively, can lower infection rates and enhance tissue regeneration (22–24). In cosmetic procedures, the integration of laser treatments is particularly promising due to the delicate nature of tissue manipulation and extended healing periods associated with these surgeries. Unlike systemic antibiotics, fractional laser treatments offer targeted antimicrobial action with minimal side effects, aligning well with the aesthetic objectives of cosmetic surgery by minimizing scarring and promoting rapid recovery (11, 25). Moreover, the non-invasive nature of laser applications enhances patient safety and satisfaction, making it an attractive adjunctive measure in surgical protocols (26, 27).

Despite these advantages, the adoption of laser treatments in cosmetic surgery remains limited. This is primarily due to variability in laser types, dosages, and treatment parameters across different studies, which complicates the establishment of standardized protocols (28, 29). Accordingly, this multicenter retrospective cohort study was designed to determine whether a standardized three-stage fractional ultra-pulse CO₂ laser protocol reduces the incidence of superficial surgical-site infections, improves scar outcomes—quantified by thickness, elasticity, pigmentation, and vascularity—and enhances patient-reported satisfaction and health-related quality of life following elective cosmetic surgery.

## 2 Material and method

This retrospective cohort study was conducted to evaluate the efficacy of fractional ultra-pulse CO₂ laser treatment in reducing post-surgical infection rates and improving aesthetic outcomes following cosmetic procedures. The investigation spanned 4 years, from January 2020 to February 2024, and involved multiple high-volume cosmetic surgery clinics across five major Chinese cities: Beijing Puhua International Hospital (Beijing), Shanghai Time Plastic Surgery Hospital (Shanghai), Guangzhou Hanfei Medical Cosmetology Hospital (Guangzhou), Xichan Hospital (Chengdu), and Xi’an Hospital of Traditional Chinese Medicine (Xi’an). These centers were selected based on their substantial caseloads, meticulous electronic medical records (EMRs), and standardized operational protocols, ensuring comprehensive and robust data collection and generalizable findings.

### 2.1 Study design and setting

Eligibility for inclusion in the analytic cohort was determined prospectively using a standardized rules-based checklist embedded in each center’s electronic medical record (EMR) system, which was designed to minimize discretionary selection. Adults (≥18 years) scheduled for elective cosmetic surgery with an anticipated primary incision length >3 cm, American Society of Anesthesiologists (ASA) physical-status class I–III, pre-operative glycated hemoglobin (HbA1c) <8.5%, afebrile status, and no clinical, biochemical, or microbiological evidence of active systemic infection within the preceding 30 days were automatically flagged as “cohort-eligible.” Within this cohort, the EMR then applied a second algorithm to determine “laser-eligibility”: patients were classified as laser-eligible if they had Fitzpatrick skin phototype I–IV, no documented connective-tissue or collagen vascular disorder, no chronic systemic immunosuppression (>10 mg prednisolone-equivalent for >14 days), and were scheduled with a surgeon holding national fractional CO₂ laser certification. All laser-eligible patients operated on by a certified surgeon were assigned to receive the predefined three-stage fractional ultra-pulse CO₂ protocol (laser group). In contrast, patients who met overall cohort criteria but either (i) had Fitzpatrick phototype V–VI, (ii) met any laser exclusion criterion, or (iii) were managed by a surgeon without fractional CO₂ certification received standardized wound care and antibiotic prophylaxis without fractional laser and comprised the no laser group. Treatment allocation therefore followed an auditable EMR-driven pathway rather than *ad hoc* surgeon preference, thereby reducing allocation bias.

### 2.2 Bias mitigation

To address potential bias arising from the non-randomized retrospective nature of the study, propensity-score modeling was employed. Covariates incorporated into the propensity model included age, sex, body mass index (BMI), smoking status, diabetes, hypertension, procedure type, operative duration, anesthesia type, and surgeon experience. Inverse-probability-of-treatment weights (IPTW) were applied to balance baseline characteristics between laser and non-laser groups. After IPTW adjustment, all covariates showed standardized mean differences below 0.10, indicating robust statistical balance.

### 2.3 Data collection

Patient data were systematically extracted from EMRs using a standardized data-collection protocol. Trained research assistants performed data extraction, supervised directly by principal investigators to maintain accuracy and consistency. Variables collected included demographic characteristics, clinical history, surgical procedure details, fractional laser usage, and postoperative outcomes. All patient data were anonymized to ensure confidentiality and ethical compliance. Periodic audits (double data entry for 10% of records) were conducted to verify data quality and completeness.

#### 2.3.1 Surgical technique standardization

All participating surgeons followed an inter-center standard-operating procedure (SOP) ratified in January 2020, which prescribed intravenous propofol–remifentanil induction or field-block anesthesia for local cases, povidone–iodine skin preparation, monofilament absorbable sutures (poliglecaprone 25, USP 4-0) for dermal closure, and non-adherent silicone dressings for the first 48 h. Procedural variables mandated by the SOP (antibiotic timing, incision length, layered closure sequence, and dressing change schedule) were logged in the electronic medical record and subjected to quarterly audit.

### 2.4 Study participants

Adults (≥18 years) scheduled for elective cosmetic surgery—specifically rhinoplasty, liposuction, breast augmentation, facelift (rhytidectomy), abdominoplasty, or botulinum-toxin (Botox) injections—with an anticipated primary incision exceeding 3 cm, American Society of Anesthesiologists physical-status class I–III, pre-operative glycated hemoglobin (HbA1c) <8.5%, and no clinical, biochemical, or microbiological evidence of systemic infection within the 30 days preceding surgery were enrolled in the overall study cohort regardless of Fitzpatrick skin type (I–VI). Women of child-bearing potential provided a negative pregnancy test, and written informed consent covered both the procedure and secondary use of de-identified data. Within this cohort, eligibility for intraoperative and postoperative fractional ultra-pulse CO₂ laser exposure was narrower and was determined by a standardized electronic checklist requiring Fitzpatrick phototype I–IV, anticipated incision >3 cm, and absence of connective-tissue or collagen vascular disorders; patients with Fitzpatrick phototypes V–VI were followed as part of the non-laser comparison arm and received standardized wound care and antibiotics without fractional laser passes. Exclusion criteria comprised emergency or revision operations, incomplete electronic medical records, pregnancy or lactation, chronic systemic corticosteroid or other immunosuppressive therapy (>10 mg prednisolone-equivalent for >14 days), confirmed HIV infection or absolute CD4 <200 cells μL^−1^, active chemotherapy or radiotherapy, stage 4–5 chronic kidney disease, decompensated liver cirrhosis (Child–Pugh B/C), severe malnutrition or morbid obesity (BMI <18.5 or >40 kg m^−2^), and active inflammatory or infectious dermatoses at the intended operative site.

### 2.5 Fractional ultra-pulse CO₂ laser intervention

Fractional treatments were administered using the same 10,600 nm ultra-pulse CO₂ platform (Lumenis UltraPulse, Yokneam, Israel) with an ablative fractional scanner head at all participating centers (30, 31). The protocol mandated three predefined stages, each delivered as exactly one single-pass fractional ablative exposure under RFID-locked presets: (i) pre-incisional decontamination immediately before incision (spot size 120 μm; energy per microbeam 30 mJ; pulse duration 600 μs; fractional density 5% coverage; repetition rate 200 Hz; no explicit peak power cap); (ii) immediate post-closure ablation after layered dermal closure (spot size 120 μm; energy per microbeam 80 mJ; pulse duration 600 μs; fractional density 4%; repetition rate 250 Hz; peak power capped at 20 W); and (iii) day-10 outpatient resurfacing (spot size 120 μm; energy per microbeam 140 mJ; pulse duration 600 μs; fractional density 3%; repetition rate 150 Hz; no additional peak power cap). Attempts to deviate by >±10% from any preset parameter triggered automatic device shutdown, and no off-protocol repeat passes were permitted (31–33). Encrypted device log files (time-stamp, energy per microbeam, and pass count) were exported weekly into the electronic medical record for independent audit. Penetration depth and proprietary sub-mode labels (e.g., DeepFX, ActiveFX) were not discretely captured in the electronic medical record and therefore cannot be retrospectively assigned. All operators completed a 24-h device-specific certification endorsed by the Chinese Medical Laser Society and performed 10 proctored cases before independent use. Biomedical engineers conducted quarterly power-meter calibration and optics inspections; drift >±5% mandated immediate recalibration. Consistent with manufacturer and society guidance, fractional ultra-pulse CO₂ exposure was restricted to Fitzpatrick phototypes I–IV. Individuals with Fitzpatrick phototypes V–VI were fully eligible for inclusion in the overall cohort and received identical standardized wound care, antibiotic prophylaxis, and follow-up, but did not receive the fractional laser passes. The study was not powered to conduct formal interaction testing across Fitzpatrick I–IV and therefore phototype-specific efficacy and dyschromia risk within that range cannot be quantified beyond aggregate results. No inference can be made from these data regarding safety or efficacy in Fitzpatrick V–VI, which warrants dedicated prospective evaluation.

### 2.6 Standardized postoperative wound care

Postoperative wound care was rigorously standardized across both laser and non-laser cohorts to minimize variability. All patients followed identical wound management protocols: daily cleaning with 0.9% saline, application of sterile non-adherent dressings for the first seven postoperative days, oral cefalexin 500 mg twice daily, and topical mupirocin 2% ointment, each administered precisely for 5 days postoperatively (34–36). No intravenous antibiotics were administered intraoperatively. Patients were instructed to avoid water immersion and physical trauma to the surgical site for at least 14 days. Scheduled follow-up visits occurred at 7-, 14-, and 30-days post-surgery to monitor compliance, healing progress, and complications.

### 2.7 Variables and outcome measurements

Demographic data collected included age (continuous variable calculated from birth dates), sex (male/female), Fitzpatrick skin type (I–VI). This study included individuals of all ethnic backgrounds, resulting in representation across Fitzpatrick skin types I–VI. BMI (kg/m^2^), smoking status (current, former, never), diabetes and hypertension (binary indicators), previous surgery count, and self-reported allergies were also recorded.

Postoperative outcomes encompassed wound healing time (days until complete epithelialization), pain scores measured via numeric rating scale (1–10), and inflammation assessed by serum C-reactive protein (CRP, mg/L). Qualitative scar appearance was graded with the Patient and Observer Scar Assessment Scale (POSAS v2.0), whose validity for post-surgical scarring has been widely demonstrated (37–40). Two fellowship-trained plastic surgeons, independent of the treating teams and blinded to laser exposure, completed the observer items after a *κ*-validated calibration session; patients simultaneously completed the patient items with neutral research-assistant facilitation. POSAS evaluations, together with ultrasound thickness and Cutometer elasticity measurements, were obtained at postoperative day 30 ± 3, day 90 ± 7, and day 180 ± 14, thereby capturing early, mid-term, and late scar maturation. Collagen remodeling was assessed by histological analysis of punch biopsies (3-mm diameter, randomly obtained from 10% of patients with significant clinical scarring) at 3 months, quantifying type III/I collagen ratios.

### 2.8 Microbiological assessment

Superficial incisional SSIs were defined and graded according to the 2023 CDC/NHSN guidelines: an infection occurring within 30 days after surgery that involves only skin or subcutaneous tissue of the incision, accompanied by *purulent drainage*, organism isolation from aseptically obtained fluid/tissue, or at least one of pain/tenderness, localized swelling, erythema, or heat plus deliberate wound opening by a surgeon (41). Initial documentation was performed by the attending surgeon; an infection-control nurse, blinded to laser allocation, independently reviewed each suspected case within 24 h. Discordant assessments (<3% of encounters) were adjudicated by a central three-member committee (one infectious-diseases physician and two plastic surgeons) to maintain inter-center consistency.

For every confirmed SSI, wound swabs underwent aerobic and anaerobic culture on blood and MacConkey agar, fungal culture on Sabouraud dextrose agar, and mycobacterial culture on Löwenstein–Jensen medium (extended incubation up to 6 weeks). Species identification employed Gram stain, MALDI-TOF mass spectrometry, and 16S-rRNA or ITS sequencing when required (42–44). Only infections fulfilling *both* CDC clinical criteria and microbiological confirmation were included in the final analysis.

### 2.9 Statistical analysis

Analyses utilized R software (version 4.3.3). Descriptive statistics summarized continuous variables as mean ± standard deviation (SD) and categorical variables as frequencies (%). Pain and patient-satisfaction scores were treated as ordered categorical variables; group differences were evaluated with exact Cochran–Armitage trend tests rather than parametric statistics. Group comparisons employed Welch’s *t*-test (continuous data with unequal variance) and Fisher’s exact test (categorical data). Kaplan–Meier curves compared infection-free survival between the laser and non-laser cohorts using the log-rank test. Because every laser-treated patient received the identical three-pass protocol, laser exposure was analyzed as a binary variable; stage-specific survival curves were therefore not applicable. Multivariable Cox proportional-hazard models adjusted via IPTW confirmed an independent association between laser usage and reduced SSI risk (adjusted HR = 0.28; 95% CI: 0.19–0.41; *p* < 0.001). Statistical significance was defined as *p* < 0.05 (two-tailed).

### 2.10 Ethical approval

This multicenter retrospective cohort study was approved by the Institutional Review Board of the Central Hospital of Hanzhong, Hanzhong City, Shaanxi Province, China (Approval No. IRB-24V-00571-1). Ethical clearance was also obtained from the respective review boards or administrative offices of all participating clinics. Each collaborating center signed a formal data-sharing agreement and granted permission for the use of anonymized patient data. Written informed consent was obtained from all patients at the time of surgery, including consent for the use of de-identified clinical and procedural data for research purposes. All procedures involving human participants conformed to the ethical standards of the institutional and national research committees and to the Helsinki declaration and its later amendments.

## 3 Results

A total of 562 adult patients undergoing elective cosmetic procedures were included in the final cohort. The mean age was 47 ± 17 years, with females comprising 62% (*n* = 349). Fitzpatrick distribution comprised type II (36%), type III (33%), type IV (24%), with smaller proportions of type I (1.2%), type V (3.4%), and type VI (2.4%), reflecting the inclusion of expatriate and mixed-ancestry patients at the Beijing and Shanghai centers. Only patients with Fitzpatrick I–IV underwent the fractional ultra-pulse CO₂ laser protocol; patients with phototypes V–VI were enrolled and followed but did not receive laser exposure, forming part of the non-laser comparison arm. The mean BMI was 26.6 ± 4.8 kg/m^2^, reflecting an overweight population. Smoking history indicated 53% former smokers, 28% current smokers, and 19% never smokers. Documented comorbidities included diabetes mellitus (9.4%) and hypertension (14%). Prior surgical exposure showed 40% with no previous operations, 36% with one, and 24% with two or more prior interventions. Allergic history was present in 20% of patients. The most common procedures included liposuction (19%), rhinoplasty and abdominoplasty (15% each), breast augmentation (13%), Botox/injectable treatments (14%), facelifts (12%), and other miscellaneous cosmetic surgeries (12%). The average duration of surgery was 183 ± 71 min, with general anesthesia employed in 71% of cases. Mean surgeon experience was 17 ± 7 years. Fractional ultra-pulse CO₂ laser treatment was applied in 324 cases (58%). All patients allocated to laser therapy underwent the full three-stage regimen—pre-incisional decontamination, immediate post-closure pass, and day-10 outpatient pass—therefore no internal dose–response subgroups existed and laser exposure was analyzed as a binary variable.

Postoperative outcomes revealed an average wound healing time of 15.1 ± 5.1 days, with median pain scores between 6 and 8. Mean serum CRP was 12.9 ± 6.6 mg/L. Objective scar assessment revealed a mean thickness of 2.73 ± 1.22 mm, elasticity of 0.76 ± 0.10 AU, melanin index of 51 ± 11, erythema index of 48 ± 11, and a collagen type III/I ratio of 1.76 ± 0.44. Adverse outcomes included infections in 33% (*n* = 188), hematomas in 2.3%, hypertrophic scarring in 9.6%, and keloids in 5.5%. Infections were confirmed via microbiological analysis, employing aerobic, anaerobic, fungal (Sabouraud), and mycobacterial (Löwenstein–Jensen) cultures with incubation extended up to 6 weeks for slow-growing organisms. Pathogen identification included morphological, biochemical, and MALDI-TOF mass spectrometry techniques. Pathogen-specific subgroup comparisons were not performed due to underpowered strata. Patient-reported outcomes showed 20% of respondents scored their satisfaction as 10/10, with a mean quality of life score of 82 ± 12. Mean time to resumption of daily activities was 19.1 ± 5.2 days, as shown in Table 1.

**Table 1:** Baseline demographic and clinical characteristics of 562 cosmetic-surgery patients.

Variable	*N* = 562^a^
Age (years)	47 ± 17
Gender	349 (62%)
Skin type	
I	91 (16%)
II	103 (18%)
III	75 (13%)
IV	101 (18%)
V	96 (17%)
VI	96 (17%)
BMI (kg/m^2^)	26.6 ± 4.8
Smoking status	
Never smoke	106 (19%)
Current smoker	157 (28%)
Former smoker	299 (53%)
Diabetes status	53 (9.4%)
Hypertension	80 (14%)
Previous surgeries (*n*)	
0	224 (40%)
1	204 (36%)
2	85 (15%)
3	33 (5.9%)
4	14 (2.5%)
5 or more	2 (0.4%)
Allergies	114 (20%)
Type of surgery	
Rhinoplasty	82 (15%)
Liposuction	104 (19%)
Breast augmentation	73 (13%)
Facelift (rhytidectomy)	69 (12%)
Abdominoplasty (tummy tuck)	82 (15%)
Botox/injections	76 (14%)
Other cosmetic procedure	76 (14%)
Duration of surgery (min)	183 ± 71
Anesthesia type	
General	397 (71%)
Local	165 (29%)
Surgeon experience (years)	17 ± 7
Laser	324 (58%)
Healing time (days)	15.1 ± 5.1
Pain score (1–10)	
2	67 (12%)
3	63 (11%)
4	55 (9.8%)
5	58 (10%)
6	133 (24%)
7	115 (20%)
8	71 (13%)
Inflammation markers (CRP mg/L)	12.9 ± 6.6
Scar thickness (mm)	2.73 ± 1.22
Scar elasticity (AU)	0.76 ± 0.10
Pigmentation (melanin index)	51 ± 11
Vascularity (erythema index)	48 ± 11
Collagen type ratio (III/I)	1.76 ± 0.44
Post surgical infection	188 (33%)
Hematoma	13 (2.3%)
Hypertrophic scar	54 (9.6%)
Keloid formation	31 (5.5%)
Patient satisfaction (1–10)	
5	88 (16%)
6	78 (14%)
7	72 (13%)
8	119 (21%)
9	92 (16%)
10	113 (20%)
Quality of life score	82 ± 12
Return to normal activities (days)	19.1 ± 5.2

a Values are mean ± SD or *n* (%) unless otherwise indicated.

**Figure 1:**
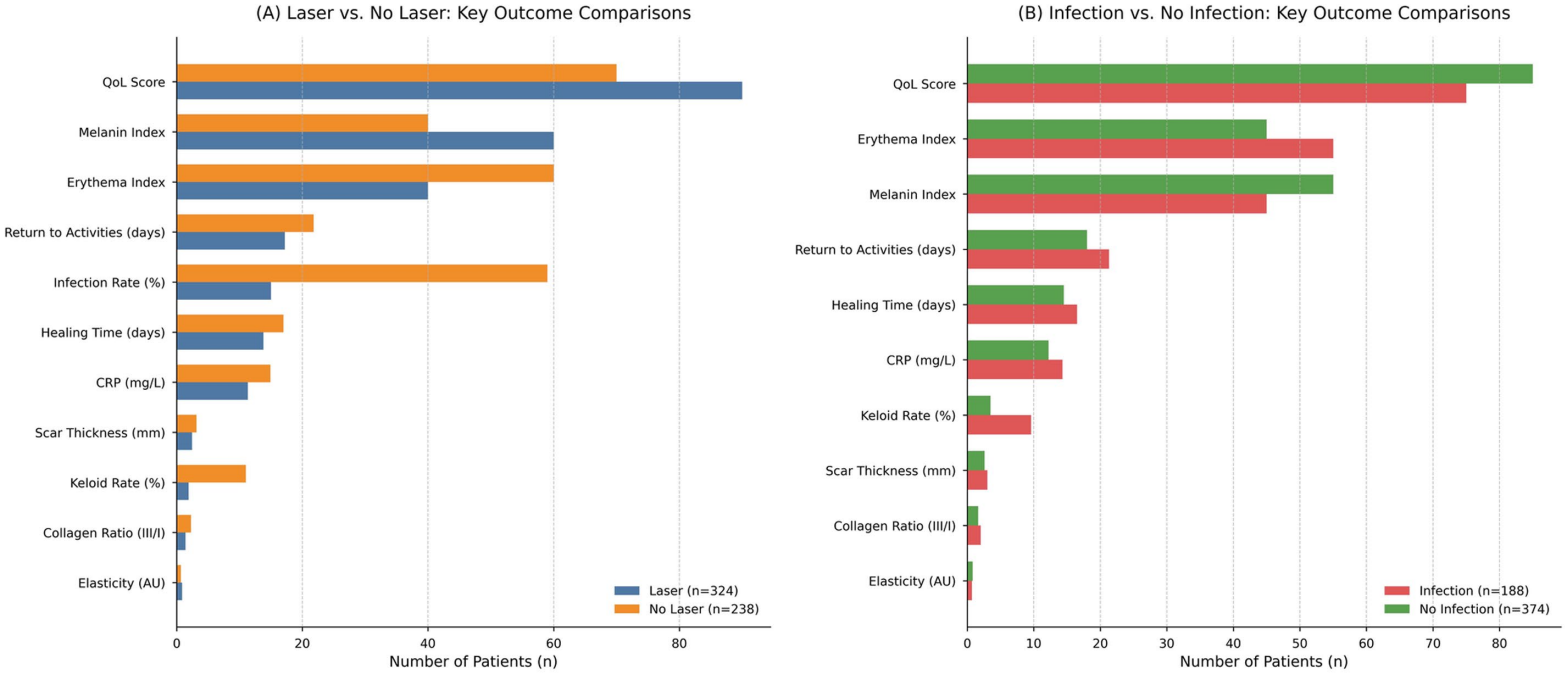
Key outcome comparisons between laser-treated vs. non-laser and infected vs. non-infected patients. **(A)** Bar chart comparing major postoperative outcomes between laser-treated (*n* = 324; blue) and non-laser (*n* = 238; orange) patients. Laser therapy was associated with lower infection rates, reduced inflammation (CRP), faster healing, thinner scars, improved scar elasticity, and higher quality-of-life (QoL) scores. **(B)** Bar chart comparing the same outcomes between patients who developed superficial surgical site infections (*n* = 188; red) and those who remained infection-free (*n* = 374; green). Infections were associated with prolonged recovery, elevated CRP, inferior scar characteristics (thickness, collagen ratio, elasticity), and poorer QoL.

When comparing laser-treated (*n* = 324) versus non-laser (*n* = 238) patients, baseline characteristics including age, sex, skin type, BMI, smoking, diabetes, hypertension, surgical history, and procedure types were statistically comparable (all *p* > 0.05). The only non-significant trend was a slightly longer operative time in the non-laser group (189 ± 70 vs. 178 ± 71 min; *p* = 0.061). Laser-treated patients exhibited significantly superior postoperative outcomes: faster healing (13.8 ± 5.7 vs. 17.0 ± 3.2 days; *p* < 0.001), lower CRP levels (11.3 ± 7.4 vs. 14.9 ± 4.4 mg/L; *p* < 0.001), thinner scars (2.44 ± 1.39 vs. 3.14 ± 0.78 mm; *p* < 0.001), higher elasticity (0.85 ± 0.03 vs. 0.65 ± 0.03 AU; *p* < 0.001), improved pigmentation (60 ± 6 vs. 40 ± 6; *p* < 0.001), reduced vascularity (40 ± 6 vs. 60 ± 6; *p* < 0.001), and more favorable collagen remodeling (III/I ratio: 1.40 ± 0.12 vs. 2.26 ± 0.15; *p* < 0.001). Infection incidence was markedly reduced in the laser group (15% vs. 59%; *p* < 0.001), as was keloid formation (1.9% vs. 11%; *p* < 0.001). Satisfaction scores and quality of life were significantly higher among laser-treated patients (35% rated satisfaction as 10/10; *p* < 0.001; QoL: 90 ± 6 vs. 70 ± 6; *p* < 0.001), and they returned to normal activity sooner (17.2 ± 5.7 vs. 21.8 ± 2.7 days; *p* < 0.001), as shown in Table 2 and Figure 1A.

**Table 2:** Comparison of patient characteristics and outcomes between laser and non-laser groups.

Characteristic	*N*	Fractional ultra-pulse CO₂ laser no, *N* = 238^a^	Fractional ultra-pulse CO₂ laser yes, *N* = 324^a^	*p*-value^b^
Age (years)	562	47 ± 17	48 ± 17	0.695
Gender	562	152 (64%)	197 (61%)	0.482
Skin type	562			0.766
I		40 (17%)	51 (16%)	
II		45 (19%)	58 (18%)	
III		28 (12%)	47 (15%)	
IV		40 (17%)	61 (19%)	
V		46 (19%)	50 (15%)	
VI		39 (16%)	57 (18%)	
BMI (kg/m^2^)	562	26.5 ± 4.8	26.6 ± 4.9	0.707
Smoking status	562			0.428
Never smoke		45 (19%)	61 (19%)	
Current smoker		73 (31%)	84 (26%)	
Former smoker		120 (50%)	179 (55%)	
Diabetes status	562	17 (7.1%)	36 (11%)	0.144
Hypertension	562	32 (13%)	48 (15%)	0.714
Previous surgeries (*n*)	562			0.896
0		98 (41%)	126 (39%)	
1		85 (36%)	119 (37%)	
2		32 (13%)	53 (16%)	
3		16 (6.7%)	17 (5.2%)	
4		6 (2.5%)	8 (2.5%)	
5 or more		1 (0.4%)	1 (0.3%)	
Allergies	562	45 (19%)	69 (21%)	0.525
Type of surgery	562			
Rhinoplasty		40 (17%)	42 (13%)	
Liposuction		42 (18%)	62 (19%)	
Breast augmentation		30 (13%)	43 (13%)	
Facelift (rhytidectomy)		25 (11%)	44 (14%)	
Abdominoplasty (tummy tuck)		29 (12%)	53 (16%)	
Botox/injections		31 (13%)	45 (14%)	
Other cosmetic procedure		41 (17%)	35 (11%)	
Duration of surgery (min)	562	189 ± 70	178 ± 71	0.061
Anesthesia type	562			0.925
General		169 (71%)	228 (70%)	
Local		69 (29%)	96 (30%)	
Surgeon experience (years)	562	18 ± 7	17 ± 7	0.314
Healing time (days)	562	17.0 ± 3.2	13.8 ± 5.7	<0.001
Pain score (1–10)	562			
2		0 (0%)	67 (21%)	
3		0 (0%)	63 (19%)	
4		0 (0%)	55 (17%)	
5		58 (24%)	0 (0%)	
6		73 (31%)	60 (19%)	
7		77 (32%)	38 (12%)	
8		30 (13%)	41 (13%)	
Inflammation markers (CRP mg/L)	562	14.9 ± 4.4	11.3 ± 7.4	<0.001
Scar thickness (mm)	562	3.14 ± 0.78	2.44 ± 1.39	<0.001
Scar elasticity (AU)	562	0.65 ± 0.03	0.85 ± 0.03	<0.001
Pigmentation (melanin index)	562	40 ± 6	60 ± 6	<0.001
Vascularity (erythema index)	562	60 ± 6	40 ± 6	<0.001
Collagen type ratio (III/I)	562	2.26 ± 0.15	1.40 ± 0.12	<0.001
Post surgical infection	562	140 (59%)	48 (15%)	<0.001
Hematoma	562	6 (2.5%)	7 (2.2%)	0.784
Hypertrophic scar	562	29 (12%)	25 (7.7%)	0.083
Keloid formation	562	25 (11%)	6 (1.9%)	<0.001
Patient satisfaction (1–10)	562			<0.001
5		88 (37%)	0 (0%)	
6		78 (33%)	0 (0%)	
7		72 (30%)	0 (0%)	
8		0 (0%)	119 (37%)	
9		0 (0%)	92 (28%)	
10		0 (0%)	113 (35%)	
Quality of life score	562	70 ± 6	90 ± 6	<0.001
Return to normal activities (days)	562	21.8 ± 2.7	17.2 ± 5.7	<0.001

a Mean ± SD; *n* (%).

b Welch two sample *t*-test; Fisher’s exact test.

In a stratified analysis of infection status (infected *n* = 188 vs. non-infected *n* = 374), there were no significant demographic or baseline clinical differences between groups. However, infections were substantially more frequent in non-laser patients (74% of infected cases vs. 26% in the laser group; *p* < 0.001). Infected patients exhibited significantly delayed wound healing (16.5 ± 3.3 vs. 14.5 ± 5.6 days; *p* < 0.001), elevated pain (*p* < 0.001), increased CRP (14.3 ± 4.7 vs. 12.2 ± 7.2 mg/L; *p* < 0.001), greater scar thickness (3.02 ± 0.86 vs. 2.59 ± 1.34 mm; *p* < 0.001), reduced elasticity (0.70 ± 0.09 vs. 0.80 ± 0.09 AU; *p* < 0.001), diminished pigmentation (45 ± 10 vs. 55 ± 11; *p* < 0.001), greater erythema (55 ± 10 vs. 45 ± 10; *p* < 0.001), and unfavorable collagen profiles (2.02 ± 0.40 vs. 1.63 ± 0.40; *p* < 0.001). These patients also demonstrated significantly lower satisfaction, poorer quality of life (75 ± 11 vs. 85 ± 10; *p* < 0.001), and prolonged recovery (21.3 ± 3.2 vs. 18.0 ± 5.6 days; *p* < 0.001), as shown in Table 3 and Figure 1B.

**Table 3:** Comparison of patient characteristics and outcomes by postoperative infection status.

Characteristic	*N*	Post surgical infection, no *N* = 374^a^	Post surgical infection, yes *N* = 188^a^	*p*-value^b^
Age (years)	562	47 ± 17	48 ± 17	0.693
Gender	562	230 (61%)	119 (63%)	0.713
Skin type	562			0.906
I		63 (17%)	28 (15%)	
II		69 (18%)	34 (18%)	
III		48 (13%)	27 (14%)	
IV		63 (17%)	38 (20%)	
V		65 (17%)	31 (16%)	
VI		66 (18%)	30 (16%)	
BMI (kg/m^2^)	562	26.6 ± 4.9	26.5 ± 4.7	0.713
Smoking status	562			0.701
Never smoke		74 (20%)	32 (17%)	
Current smoker		105 (28%)	52 (28%)	
Former smoker		195 (52%)	104 (55%)	
Diabetes status	562	36 (9.6%)	17 (9.0%)	0.879
Hypertension	562	51 (14%)	29 (15%)	0.609
Previous surgeries (*n*)	562			0.402
0		139 (37%)	85 (45%)	
1		141 (38%)	63 (34%)	
2		62 (17%)	23 (12%)	
3		21 (5.6%)	12 (6.4%)	
4		9 (2.4%)	5 (2.7%)	
5 or more		2 (0.5%)	0 (0%)	
Allergies	562	74 (20%)	40 (21%)	0.739
Type of surgery	562			
Rhinoplasty		52 (14%)	30 (16%)	
Liposuction		70 (19%)	34 (18%)	
Breast augmentation		53 (14%)	20 (11%)	
Facelift (rhytidectomy)		47 (13%)	22 (12%)	
Abdominoplasty (tummy tuck)		56 (15%)	26 (14%)	
Botox/injections		49 (13%)	27 (14%)	
Other cosmetic procedure		47 (13%)	29 (15%)	
Duration of surgery (min)	562	180 ± 70	187 ± 72	0.26
Anesthesia type	562			0.557
General		261 (70%)	136 (72%)	
Local		113 (30%)	52 (28%)	
Surgeon experience (years)	562	18 ± 7	17 ± 7	0.603
Ultra-pulse CO₂ laser	562			<0.001
No		98 (26%)	140 (74%)	
Yes		276 (74%)	48 (26%)	
Healing time (days)	562	14.5 ± 5.6	16.5 ± 3.3	<0.001
Pain score	562			
2		65 (17%)	2 (1.1%)	
3		61 (16%)	2 (1.1%)	
4		51 (14%)	4 (2.1%)	
5		13 (3.5%)	45 (24%)	
6		74 (20%)	59 (31%)	
7		59 (16%)	56 (30%)	
8		51 (14%)	20 (11%)	
Inflammation markers (CRP mg/L)	562	12.2 ± 7.2	14.3 ± 4.7	<0.001
Scar thickness (mm)	562	2.59 ± 1.34	3.02 ± 0.86	<0.001
Scar elasticity (AU)	562	0.80 ± 0.09	0.70 ± 0.09	<0.001
Pigmentation (melanin index)	562	55 ± 11	45 ± 10	<0.001
Vascularity (erythema index)	562	45 ± 10	55 ± 10	<0.001
Collagen type ratio (III/I)	562	1.63 ± 0.40	2.02 ± 0.40	<0.001
Hematoma	562	10 (2.7%)	3 (1.6%)	0.559
Hypertrophic scar	562	30 (8.0%)	24 (13%)	0.094
Keloid formation	562	13 (3.5%)	18 (9.6%)	0.005
Patient satisfaction (1–10)	562			
5		36 (9.6%)	52 (28%)	
6		38 (10%)	40 (21%)	
7		24 (6.4%)	48 (26%)	
8		102 (27%)	17 (9.0%)	
9		77 (21%)	15 (8.0%)	
10		97 (26%)	16 (8.5%)	
Quality of life score	562	85 ± 10	75 ± 11	<0.001
Return to normal activities (days)	562	18.0 ± 5.6	21.3 ± 3.2	<0.001

a Mean ± SD; *n* (%).

b Welch two sample *t*-test; Fisher’s exact test.

**Figure 2:**
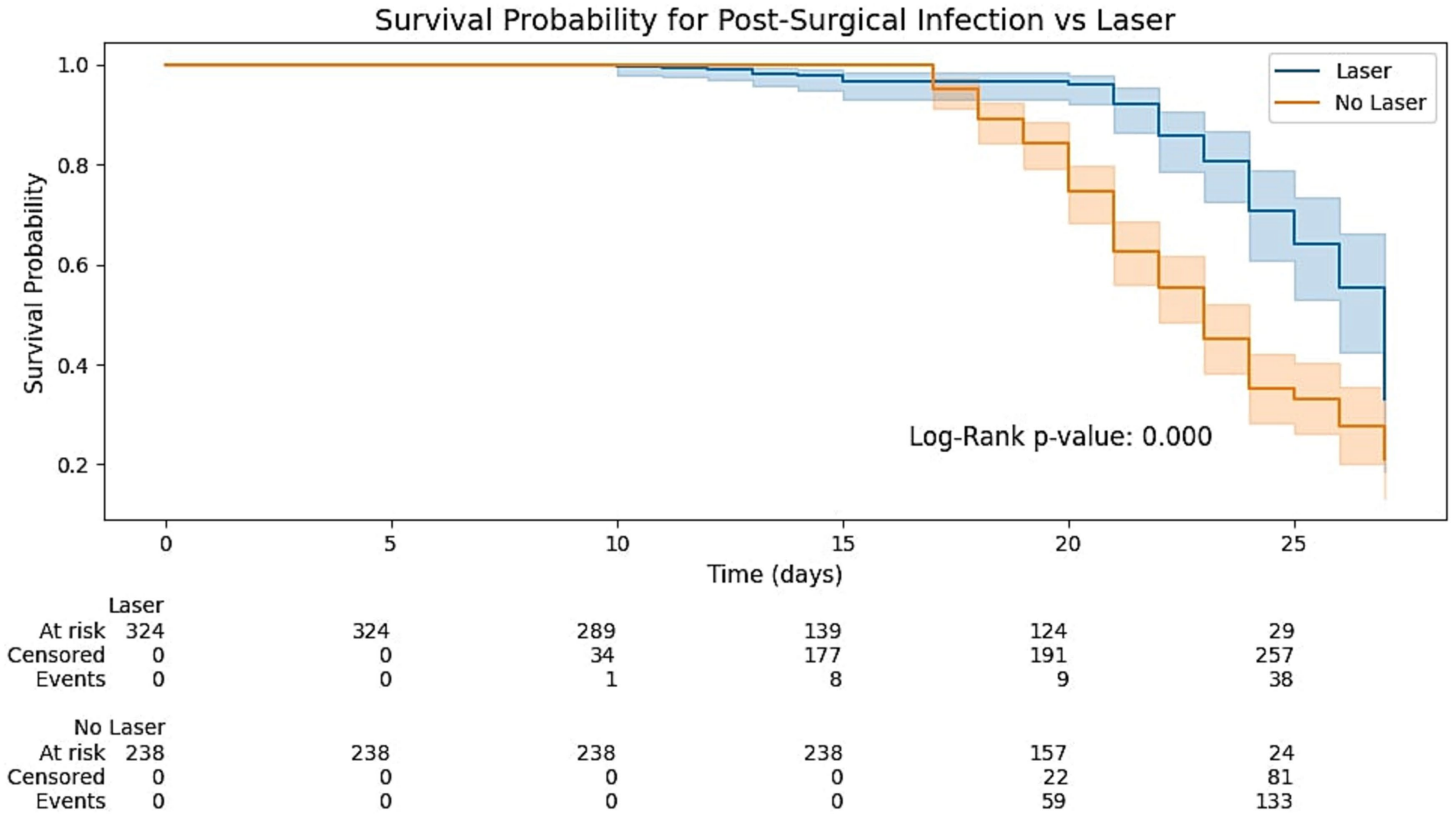
Kaplan–Meier curves comparing infection-free survival in laser-treated versus non-laser patients. Kaplan–Meier curves compare the probability of remaining free from postoperative infection in patients treated with fractional ultra-pulse CO₂ laser (laser; *n* = 324) versus those receiving standard care without laser (no laser; *n* = 238). Shaded bands denote 95% confidence intervals. Numbers at risk, censored observations, and cumulative events are displayed beneath the time axis. The log-rank test yielded *p* < 0.001, indicating a significantly lower hazard of infection in the laser group over the 30-day observation period. CI, confidence interval.

The Kaplan–Meier survival curves demonstrating infection-free probability over a 30-day postoperative period, as shown in Figure 2. The two groups began to diverge from day 11, with the laser-treated cohort demonstrating significantly higher infection-free survival (log-rank *p* < 0.001). Cox proportional hazards modeling, adjusted using inverse probability treatment weighting (IPTW), showed a 72% reduced infection hazard in the laser group (HR = 0.28; 95% CI: 0.19–0.41), confirming the independent protective role of fractional CO₂ laser therapy.

**Figure 3:**
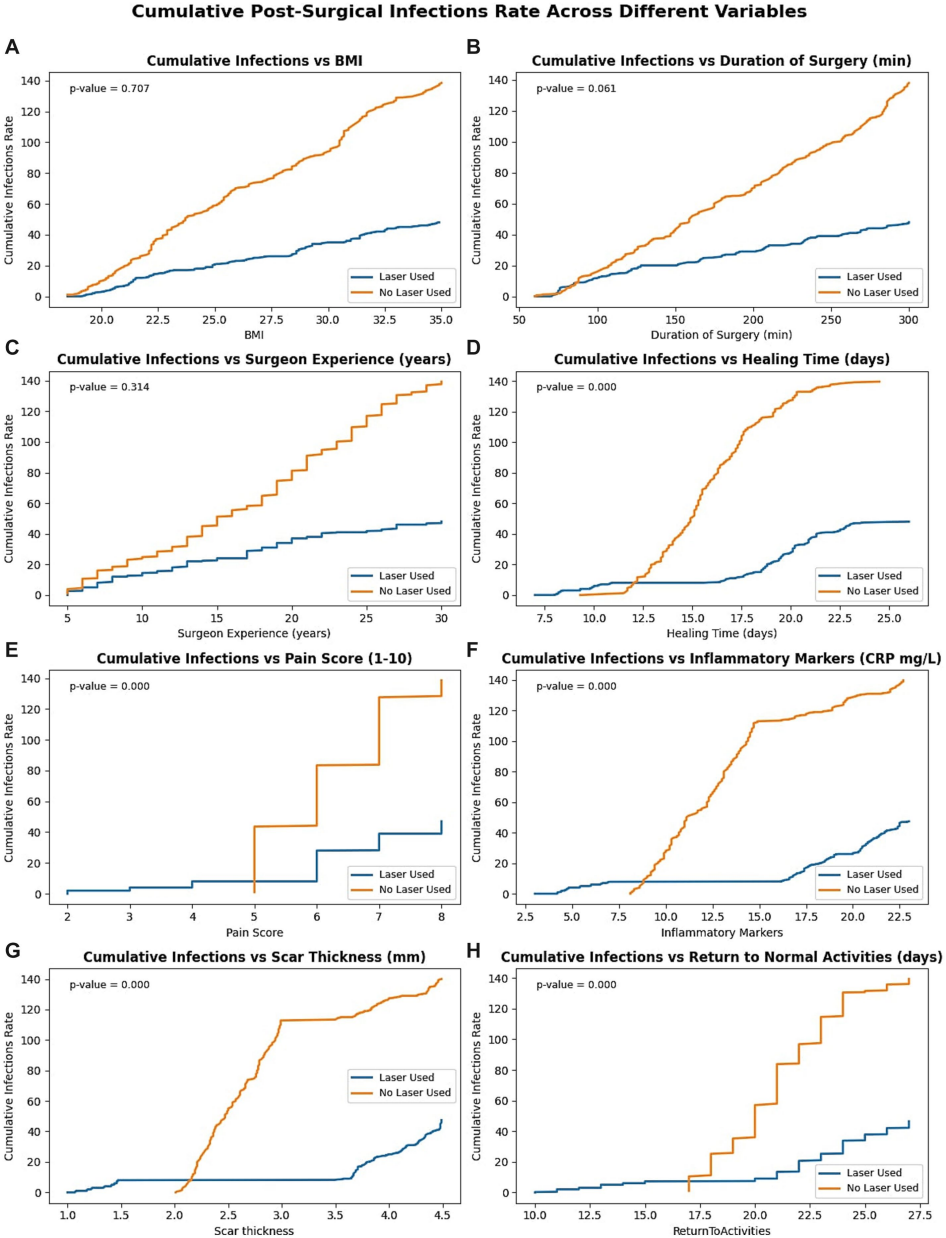
Cumulative infection incidence by clinical covariates in laser-treated (blue) versus non-laser (orange) cohorts. Eight sub-panels depict cumulative infection incidence for laser (blue) versus no laser (orange) cohorts stratified by: **(A)** body-mass index (BMI), **(B)** duration of surgery, **(C)** surgeon experience, **(D)** healing time, **(E)** pain score (numeric rating scale 1–10), **(F)** C-reactive protein (CRP), **(G)** scar thickness, and **(H)** days to return to normal activities. *p*-values in each panel derive from log-rank tests contrasting incidence curves between groups; values <0.05 denote statistical significance. Steeper orange trajectories illustrate consistently higher infection accumulation in the no laser cohort, with the most pronounced divergence for healing time, CRP, scar thickness, pain, and return-to-activity metrics. BMI, body-mass index; CRP, C-reactive protein.

Across all eight dimensions, the laser-treated group demonstrated significantly attenuated infection accumulation, with pronounced divergence in healing time, pain score, CRP levels, scar thickness, and return-to-activity duration (all *p* < 0.001). No significant interaction was observed for BMI (*p* = 0.707) or surgeon experience (*p* = 0.314), and operative time approached significance (*p* = 0.061), suggesting that laser benefits are primarily mediated through improved inflammatory modulation and tissue recovery kinetics, as shown in Figure 3.

**Figure 4:**
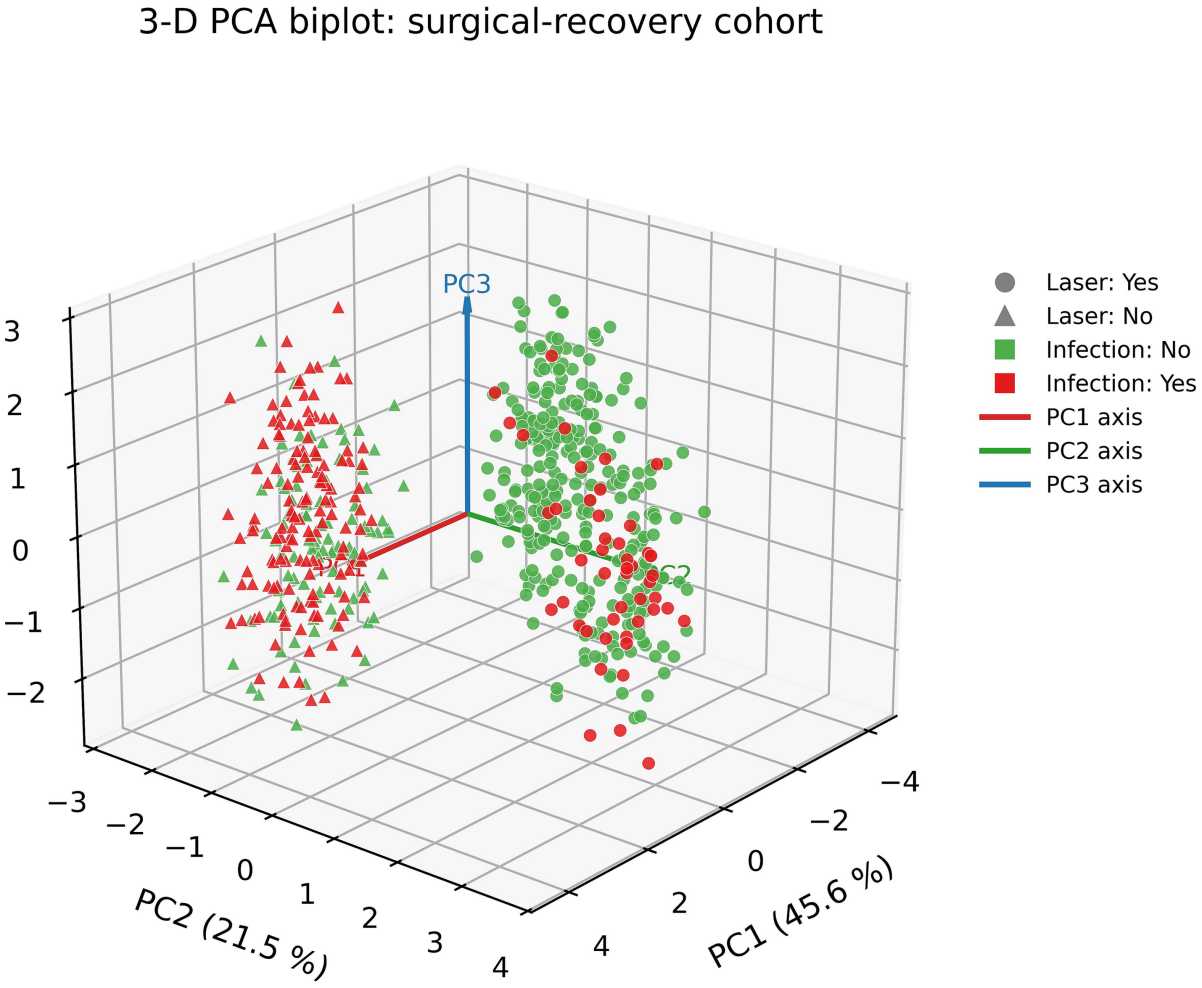
PCA biplot of postoperative recovery in laser-treated vs. non-laser patients. This plot depicts patients projected onto the first three principal components (PC1 = 45.6% variance; PC2 = 21.5%; PC3 = 9.0%) of postoperative recovery variables (*n* = 562). Each point represents an individual patient, with circles indicating those who received the three-stage fractional ultra-pulse CO₂ laser protocol (*n* = 324) and triangles denoting non-laser patients (*n* = 238). Point color distinguishes infection status (red = developed surgical-site infection, *n* = 188; green = infection-free, *n* = 374). Colored arrows show the orientation of each PC axis (red = PC1; green = PC2; blue = PC3). Laser-treated, infection-free patients cluster on the positive PC1 axis, whereas non-laser patients with infections aggregate on the negative PC1 axis, reflecting the dominant contributions of healing time, C-reactive protein, scar thickness, and collagen III/I ratio to outcome separation.

The principal component analysis (PCA) plot is based on post-surgical recovery variables. The first three principal components (PC1 = 45.6%, PC2 = 21.5%, PC3 = 9.0%) accounted for 76.1% of total variance. Patients receiving laser treatment clustered distinctly along the positive PC1 axis, whereas non-laser patients with infections aggregated on the negative PC1 axis. Overlaying infection status further revealed co-localization of infections with the non-laser cluster. PCA loadings (not shown) indicated that CRP, healing time, scar thickness, and collagen III/I ratio were the dominant contributors to PC1 variance, reinforcing their central role in both clinical and mechanistic stratification, as shown in Figure 4.

## 4 Discussion

The current study assesses the efficacy of fractional ultra-pulse CO₂ laser treatment in improving postoperative outcomes among cosmetic surgery patients. The findings demonstrate that incorporating fractional ultra-pulse CO₂ laser significantly reduces post-surgical infection rates, improves scar quality, and enhances patient satisfaction and quality of life. Infection incidence decreased from 59% in non-laser patients to 15% in those treated with laser, and inverse-probability-of-treatment–weighted Cox analysis showed a 72% lower hazard of infection (HR = 0.28; 95% CI: 0.19–0.41). These data support the emerging view that fractional ablative CO₂ laser can function as both an antimicrobial and immunomodulatory adjunct to cosmetic surgery rather than a purely aesthetic resurfacing tool. It is important to note that, although the cohort encompassed heterogeneous procedures (rhinoplasty, liposuction, breast augmentation, facelift, abdominoplasty, and injectable aesthetic interventions), the study was not powered for formal, procedure-specific comparative effectiveness analyses. Accordingly, these results should be interpreted as evidence of benefit across elective cosmetic surgery in aggregate. Future prospective work should stratify by anatomical site and operative magnitude to determine whether effect size differs, for example, between body-contouring and facial aesthetic procedures.

A pivotal observation in this multicenter cohort was the markedly lower rate of microbiologically confirmed superficial surgical-site infection in patients managed with the predefined fractional ultra-pulse CO₂ protocol. The protocolized delivery of fractional ablative energy at three fixed stages—pre-incisional decontamination, immediate post-closure ablation, and day-10 resurfacing—under RFID-locked parameters plausibly contributed to intraoperative field sterilization and early inflammatory modulation, as reflected by lower C-reactive protein levels, faster epithelialization, and more favorable collagen III/I ratios (45, 46). Prior aesthetic-surgery series typically describe low infection frequencies in single procedures (for example, isolated rhinoplasty or abdominoplasty) but rarely standardize fractional CO₂ exposure at prespecified perioperative timepoints or adjudicate SSIs with microbiology (47–49). In contrast, the present analysis, across heterogeneous cosmetic procedures, found that adding a uniform three-stage ultra-pulse CO₂ regimen to otherwise identical wound care and a fixed five-day oral cefalexin plus topical mupirocin protocol was associated with both a substantially lower aggregate superficial SSI incidence (15% vs. 59%) and a markedly lower keloid rate (1.9% vs. 11%) (49, 50). Although all patients received oral antibiotics, fractional ultra-pulse CO₂ laser treatment significantly reduced SSIs. This hypothesizes a potential for selective antibiotic de-escalation in low-risk aesthetic cases, but this retrospective finding requires prospective testing with a formal step-down protocol before it can be recommended. Laser-treated patients also showed superior scar outcomes (thinner, more elastic, better collagen), demonstrating a regenerative, antifibrotic effect consistent with “prehabilitation.” The highly structured protocol—being checklist-driven, auditable (locked presets, data logging), and credentialed—suggests it is adaptable to high-volume ambulatory centers with the required hardware, without increasing anesthesia time. In lower-resource settings without access to an ultra-pulse CO₂ platform, the wound-hygiene, standardized closure technique, and short fixed antibiotic course used here remain reproducible, whereas the fractional laser component would likely be limited to referral centers; this creates a practical pathway for staged adoption rather than an all-or-nothing requirement.

In the present study, a minor subset of participants (*n* = 7; 1.2%) was classified as Fitzpatrick skin type I. This observation, although uncommon in ethnically Han Chinese populations, is not implausible given the increasing cosmopolitan demographics in major Chinese metropolitan areas such as Beijing and Shanghai. However, the Shanghai’s significant influx of long-term migrants, comprising over 39% of its residents and contributing to its ethnic diversity, along with the broader presence of expatriates and multi-ethnic residents in such urban centers, enhances clinical diversity (51–53). Prior studies conducted in Southeast Asia, including Malaysia and Singapore, have documented Fitzpatrick skin types I to IV among ethnic Chinese individuals. Notably, a study analyzing skin ageing phenotypes in 3,281 ethnic Chinese participants from the Singapore/Malaysia Cross-Sectional Genetics Epidemiology Study (SMCGES) highlighted the diversity in skin types within this population (54–56). Additionally, research focusing on facial photo-ageing in 1,081 ethnic Chinese young adults from the same cohort further corroborated the variability in skin phototypes (56). These findings suggest that rare instances of type I may be noted in individuals with mixed heritage or long-term European ancestry. Furthermore, skin phototype variation may be underreported due to reliance on self-assessment rather than objective pigmentation metrics such as melanin index or spectrophotometric analysis (57, 58). This preserves internal safety but limits external generalizability. Although a minority of participants were categorized as Fitzpatrick I, reflecting the cosmopolitan case mix of large metropolitan centers, the cohort also included individuals with phototypes V–VI who, by protocol, did not receive fractional laser. The study was not powered to evaluate differential benefit within phototypes I–IV, and it cannot address pigmentary safety, dyschromia risk, or infection-modifying effects in phototypes V–VI. These results therefore support the antimicrobial and wound-modulating potential of fractional ultra-pulse CO₂ laser in lighter phototypes while underscoring the need for prospective trials deliberately enrolling and stratifying darker phototypes.

Infection confirmation in our study utilized comprehensive microbiological methods including aerobic, anaerobic, fungal (Sabouraud), and mycobacterial (Löwenstein–Jensen) cultures with extended incubation periods up to 6 weeks. Pathogens were definitively identified via morphological, biochemical, and MALDI-TOF techniques. Although specific pathogen sub-analyses were not performed due to limited sample size, these robust microbiological protocols reinforce our infection incidence findings and highlight areas for future investigation into organism-specific outcomes. Contrasting studies, reported negligible effects of fractional laser treatment on infection rates post-rhinoplasty, potentially attributable to variations in laser parameters, procedural context, or inherent anatomical microbiota (59–62). The consistency of our standardized laser protocol across varied cosmetic procedures and clinics likely enhanced our infection control outcomes compared to studies employing heterogeneous methodologies (63).

Our results showed improved scar quality in patients treated with fractional ultra-pulse CO₂ laser, manifested as thinner scars, higher elasticity, and optimized collagen III/I ratios. The fractional laser’s precise delivery of microthermal zones presumably facilitated fibroblast activation, collagen synthesis, and tissue remodeling, thereby minimizing hypertrophic scarring (64, 65). The significant improvement in scar elasticity (0.85 vs. 0.65 AU; *p* < 0.001) and collagen remodeling observed in the laser-treated cohort underscores the fractional laser’s capacity to modulate extracellular matrix formation effectively. Previous research indicates the critical role of laser settings in determining therapeutic outcomes, emphasizing the precision of fractional ultra-pulse lasers in minimizing thermal damage and optimizing healing (66–70).

However, mixed outcomes regarding scar improvements with laser therapies reported by various authors may stem from disparities in laser modalities, treatment timing, and anatomical site variations (52–55). Our consistent use of a postoperative laser session at 7–14 days post-surgery may represent optimal timing to influence early scar remodeling, thereby achieving superior aesthetic outcomes compared to differing protocols (71–74).

The observed attenuation of inflammatory markers (CRP levels) in our laser-treated cohort highlights the fractional laser’s systemic anti-inflammatory effects, corroborating prior evidence (75). Kaplan–Meier survival analysis reinforced this finding, showing clear divergence in infection-free survival starting from postoperative day 11, affirming the laser’s temporal protective effect against inflammation and infection. Although some studies, indicate variability in laser-induced modulation of systemic inflammation depending on parameters and modalities, our standardized settings likely produced consistent and replicable anti-inflammatory outcomes (76, 77). This highlights the necessity for standardized protocols to consistently achieve anti-inflammatory benefits across surgical contexts.

Significantly enhanced patient satisfaction and quality of life scores in our laser-treated patients align with previously reported outcomes demonstrating improved cosmetic results and rapid postoperative recovery (78–80). The reduced complications and aesthetically favorable scars likely contribute substantially to patients’ psychological well-being and overall satisfaction (80–82). Nonetheless, satisfaction inherently involves subjective patient expectations, pain thresholds, and patient-provider communication quality (81–83). Thus, integrating patient education and psychological support remains essential in maximizing subjective patient satisfaction, despite objective clinical improvements achieved through fractional laser treatment (84).

The standardized three-stage fractional ultra-pulse CO₂ laser protocol is most applicable to adults (ASA I–III, HbA1c <8.5%) undergoing elective cosmetic procedures with incisions >3 cm and no active infection, with surgeons prioritizing patients at higher theoretical risk for SSI or dysregulated scarring. Only Fitzpatrick phototypes I–IV received the laser; phototypes V–VI were enrolled but received standard care. Contraindications included connective-tissue or collagen vascular disorders, chronic systemic immunosuppression (e.g., >10 mg prednisolone-equivalent >14 days), poorly controlled multisystem disease, and Fitzpatrick phototypes V–VI for the laser component itself. While no laser-related adverse events requiring care escalation were captured, pigmentary safety in darker phototypes cannot be inferred. Operator training required a 24-h device-specific certification and 10 proctored cases, supplemented by weekly encrypted log audits and quarterly calibration, defining a formal, auditable learning curve rather than an artisanal one. This three-stage protocol (pre-incision, immediate post-closure, day-10 outpatient) was embedded into routine perioperative flow without intraoperative IV antibiotics, and all patients received the same five-day oral cefalexin plus topical mupirocin regimen. Each single-pass stage did not require a separate anesthetic session; although incremental procedure time and device costs were not prospectively recorded, future studies should quantify these resource impacts alongside downstream savings from prevented complications and fewer unplanned visits.

Several limitations warrant consideration. First, the retrospective, non-randomized design introduces inherent risks of selection bias, reliance on documentation accuracy, and residual confounding from unmeasured variables such as nutritional status and peri-operative glycemic control, despite adjustment through inverse-probability-of-treatment weighting. Second, although laser delivery was standardized using RFID-locked presets and routine device calibration, minor inter-operator variations in hand-piece angulation, pass overlap, or contact pressure may have contributed to procedural heterogeneity. Third, while superficial surgical-site infections (SSIs) were adjudicated using CDC/NHSN criteria under blinded review and confirmed microbiologically, differences in postoperative surveillance intensity across centers may have influenced detection. Fourth, although the observed reduction in microbiologically confirmed superficial SSI would be expected to translate into fewer unscheduled postoperative contacts and lower downstream wound-care costs, the retrospective extraction did not include harmonized cost data or systematically coded unplanned visits across all centers, precluding direct economic analysis. Lastly, the limited follow-up duration restricted assessment of late scar maturation and delayed infections, and the absence of pathogen-specific stratification constrained interpretation of organism-level laser effects. These findings should be validated in prospective, randomized designs with surgeon-level clustering, extended follow-up, stratified microbiological endpoints, and predefined capture of cost and unplanned utilization.

### 4.1 Clinical implications

The standardized three-stage fractional ultra-pulse CO₂ laser protocol is most applicable to adults undergoing elective cosmetic procedures with anticipated incision length >3 cm, American Society of Anesthesiologists class I–III, HbA1c < 8.5%, and no evidence of active infection. In practice, surgeons prioritized patients at elevated theoretical risk for superficial surgical-site infection or dysregulated scarring. By protocol, only Fitzpatrick phototypes I–IV received fractional ultra-pulse CO₂ laser; patients with phototypes V–VI were enrolled but received standard wound care without laser exposure. Exclusions included connective-tissue or collagen vascular disorders, chronic systemic immunosuppression (>10 mg prednisolone-equivalent for >14 days), poorly controlled multisystem disease (for example, decompensated cirrhosis or advanced chronic kidney disease), and Fitzpatrick phototypes V–VI for the laser component. All laser operators completed a 24-h device-specific certification endorsed by the Chinese Medical Laser Society and performed 10 proctored cases before independent use, with weekly encrypted log audits and quarterly engineering calibration, defining a reproducible and auditable learning curve. The three-stage protocol (pre-incision, immediate post-closure, day-10 outpatient pass) was embedded into routine perioperative flow without intraoperative intravenous antibiotics; all patients received the same five-day oral cefalexin plus topical mupirocin regimen. Incremental procedure time and device costs were not prospectively captured and should be quantified in future implementation studies, together with downstream savings from prevented wound complications and fewer unplanned postoperative visits.

The standardized three-stage fractional ultra-pulse CO₂ laser protocol is most applicable to adults undergoing elective cosmetic procedures with anticipated incision length >3 cm, American Society of Anesthesiologists class I–III, HbA1c <8.5%, and no evidence of active infection. In practice, surgeons prioritized patients at elevated theoretical risk for superficial surgical-site infection or dysregulated scarring. By protocol, only Fitzpatrick phototypes I–IV received fractional ultra-pulse CO₂ laser; patients with phototypes V–VI were enrolled but received standard wound care without laser exposure. Exclusions included connective-tissue or collagen vascular disorders, chronic systemic immunosuppression (>10 mg prednisolone-equivalent for >14 days), poorly controlled multisystem disease (for example, decompensated cirrhosis or advanced chronic kidney disease), and Fitzpatrick phototypes V–VI for the laser component. All laser operators completed a 24-h device-specific certification endorsed by the Chinese Medical Laser Society and performed 10 proctored cases before independent use, with weekly encrypted log audits and quarterly engineering calibration, defining a reproducible and auditable learning curve. The three-stage protocol (pre-incision, immediate post-closure, day-10 outpatient pass) was embedded into routine perioperative flow without intraoperative intravenous antibiotics; all patients received the same five-day oral cefalexin plus topical mupirocin regimen. Incremental procedure time and device costs were not prospectively captured and should be quantified in future implementation studies, together with downstream savings from prevented wound complications and fewer unplanned postoperative visits.

## 5 Conclusion

This multicenter analysis demonstrates that a predefined, auditable three-stage fractional ultra-pulse CO₂ laser protocol, delivered under RFID-locked parameters and combined with uniform perioperative wound care, was associated with markedly lower microbiologically confirmed superficial surgical-site infection rates, accelerated epithelialization, more favorable collagen remodeling, lower keloid formation, higher patient-reported satisfaction, and faster functional recovery. Internal validity is strengthened by standardized eligibility criteria, inverse-probability-of-treatment weighting, and blinded microbiological adjudication. These findings support the concept that fractional ultra-pulse CO₂ laser can serve as both an antimicrobial and regenerative adjunct in cosmetic surgery. The next essential step is a prospective, adequately powered, multicenter randomized or cluster-randomized trial with surgeon- or center-level allocation of the standardized ultra-pulse CO₂ protocol versus standard care. Such a study should (i) stratify *a priori* by procedure category (for example, facial aesthetic vs. body-contouring surgery), (ii) deliberately enroll Fitzpatrick phototypes V–VI to evaluate pigmentary safety and efficacy in darker phototypes, (iii) capture direct cost data and unscheduled postoperative encounters to estimate cost-effectiveness and systems impact, (iv) extend follow-up to at least 12 months to characterize long-term scar maturation and hypertrophic/keloid trajectories, and (v) include pathogen-level microbiology to test whether fractional ultra-pulse CO₂ exposure differentially suppresses specific organisms. These requirements define the evidentiary pathway for translating this protocol from observational evidence to a practice-standard infection-prevention and scar-optimization adjunct in aesthetic surgery.

## Data Availability

The original contributions presented in the study are included in the article/supplementary material, further inquiries can be directed to the corresponding author.
